# P-1721. Evaluation of lateral flow assay for diagnosis of chronic pulmonary aspergillosis

**DOI:** 10.1093/ofid/ofaf695.1892

**Published:** 2026-01-11

**Authors:** Indra Shekhar prasad, Immaculata Xess

**Affiliations:** All India Institute of Medical Sciences, New Delhi, Delhi, India; All India Institute of Medical Sciences, New Delhi, New Delhi, Delhi, India

## Abstract

**Background:**

CPA is a chronic lung disease affecting many people globally, mainly those with previous TB. It shows high mortality if untreated. Diagnosis is challenging due to non specific symptoms and limitations of traditional methods. In high TB burden countries like India, where post TB cavitations are common, CPA is a growing concern. LFA meet this need by being rapid, equipment free, and suitable for resource limited settings enabling early detection and timely treatment to reduce disease burden and improve outcome.

**Figure:**
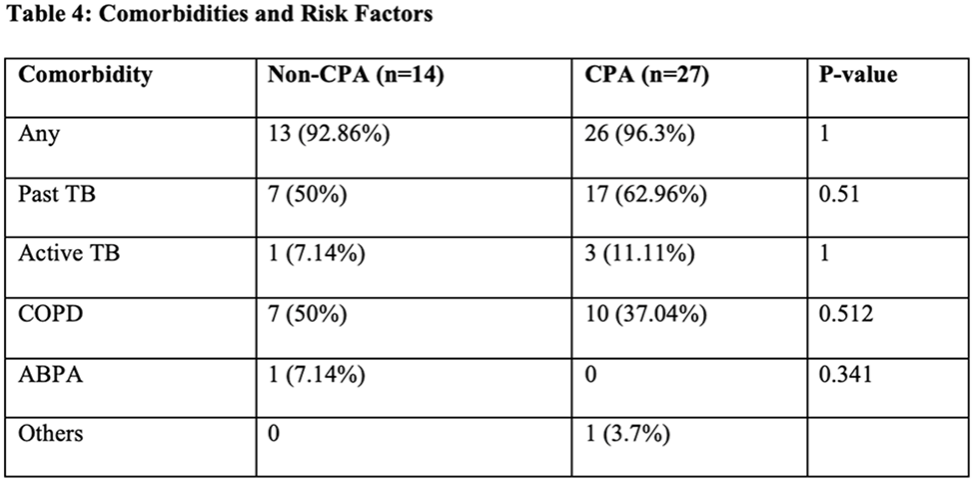

**Figure:**
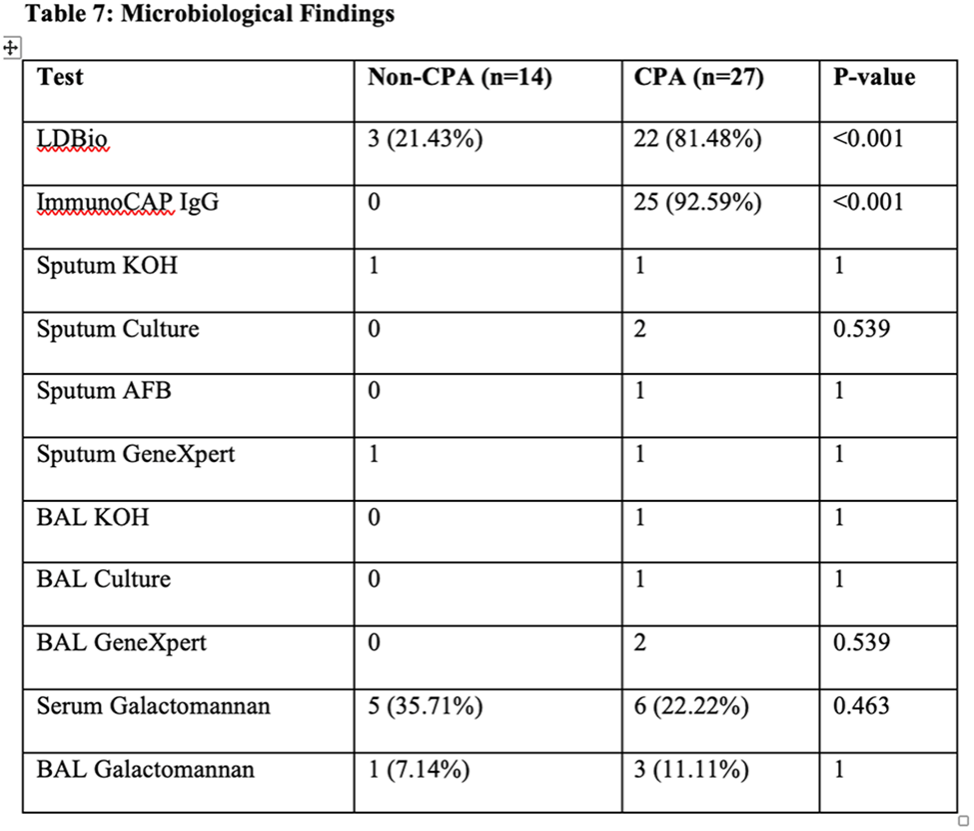
Comorbidities and risk factors for chronic pulmonary aspergillosis Microbiological evaluation of all the subjects in CPA and non-CPA groups

**Methods:**

This a prospective observational study was conducted at AIIMS, New Delhi, India from June 2020 to July 2022 to evaluate the diagnostic performance of the lateral flow assay for CPA. Patient aged > or equal to age 14 years with clinical and radiological features suggestive of CPA were included, while those on prolonged antifungal therapy, prior invasive aspergillosis were excluded. All participants underwent comprehensive clinical, radiological, and microbiological assessments. The primary outcome was the sensitivity and specificity of LFA compared to ELISA. Secondary outcome include longitudinal assessment of patients to evaluate the monitoring potential of LFA. Data were analyzed using stata version 14.1, with statistical tests considering p-value < 0.05 as significant.

**Figure:**
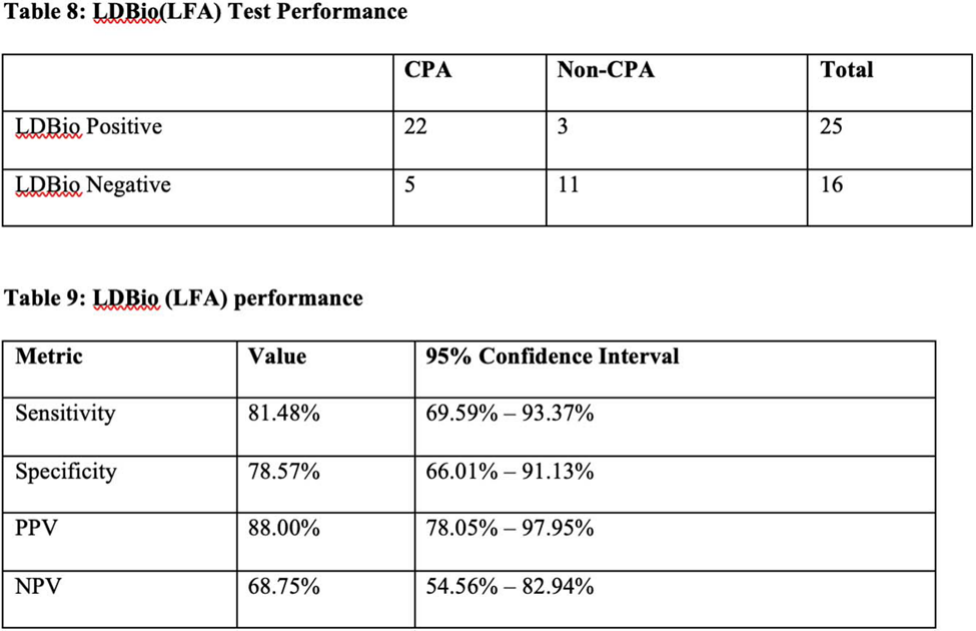
Performance of Lateral flow assay for diagnosis of CPA

**Figure:**
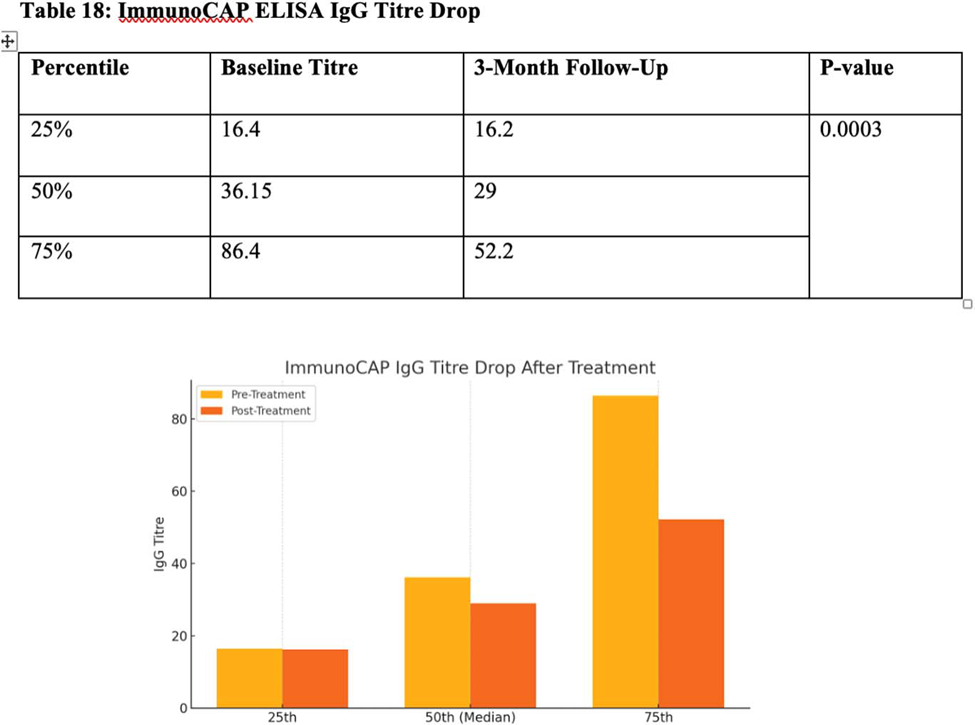
Performance of ImmunoCap ELISA for monitoring treatment response in CPA patients

**Results:**

Out of 41 included , and 27 were diagnosed with Chronic Pulmonary Aspergillosis based on clinical, radiological and microbiological evidence. Common risk factors included past TB and COPD. LFA test showed good diagnostic performance with a sensitivity of 81.48% and specificity of 78.57%. PPV remained high as 88% and NPV was moderate (68.75%). Around 74% (30 patients) were followed up. However, LFA role in treatment monitoring is limited—100% (17/17) of patients remained LFA-positive even after 3 months of antifungal therapy, (p=0.157). In contrast, ImmunoCAP ELISA titres showed significant reduction post-treatment (p=0.0003) and may serve better for monitoring.

**Conclusion:**

The lateral flow assay is a valuable rapid screening tool for CPA, particularly in a resource limited and high TB burden settings. It offers good sensitivity and ease of use, making it suitable for early detection and single sample testing. However ELISA remains superior for diagnosis and monitoring treatment response.

**Disclosures:**

All Authors: No reported disclosures

